# Combined ultrasound-guided cutting-needle biopsy and standard pleural biopsy for diagnosis of malignant pleural effusions

**DOI:** 10.1186/s12890-016-0318-x

**Published:** 2016-11-17

**Authors:** Jinlin Wang, Xinghua Zhou, Xiaohong Xie, Qing Tang, Panxiao Shen, Yunxiang Zeng

**Affiliations:** 1 Department of Respiratory Disease, The State Key Laboratory of Respiratory Disease, China Clinical Research Centre for Respiratory Disease, Guangzhou Institute of Respiratory Disease, First Affiliated Hospital of Guangzhou Medical University, 151 Yanjiang Rd, Guangzhou, 510120 Guangdong Province China; 2 Department of Ultrasound, First Affiliated Hospital of Guangzhou Medical University, Guangzhou, China

**Keywords:** Ultrasound, Cutting-needle biopsy, Pleural biopsy, Pleural effusion

## Abstract

**Background:**

The most efficient approach to diagnose malignant pleural effusions (MPEs) is still controversial and uncertain. This study aimed to evaluate the utility of a combined approach using ultrasound (US)-guided cutting-needle biopsy (CNB) and standard pleural biopsy (SPB) for diagnosing MPE.

**Methods:**

Pleural effusions were collected from 172 patients for biochemical and microbiological analyses. US-guided CNB and SPB were performed in the same operation sequentially to obtain specimens for histological analysis.

**Results:**

US-guided CNB and SPB procedures provided adequate material for histological analysis in 90.7 and 93.0% of cases, respectively, while a combination of the 2 techniques was in 96.5% of cases. The sensitivity, specificity, positive-predictive value (PPV), negative-predictive value (NPV) and diagnostic accuracy of US-guided CNB versus SPB were: 51.2 vs 63.4%, 100 vs 100%, 100 vs 100%, 64.9 vs 72.2% and 74.4 vs 81.3%, respectively. When CNB was combined with SPB, the corresponding values were 88.6, 100, 100, 88.6 and 93.9%, respectively. Whereas sensitivity, NPV and diagnostic accuracy were not significantly different between CNB and SPB, the combination of CNB and SPB significantly improved the sensitivity, NPV and diagnostic accuracy versus each technique alone (*p* < 0.05). Significant pain (eight patients), moderate haemoptysis (two patients) and chest wall haematomas (two patients) were observed following CNB, while syncope (four patients) and a slight pneumothorax (four patients) were observed following SPB.

**Conclusions:**

Use of a combination of US-guided CNB and SPB afforded a high sensitivity to diagnose MPEs, it is a convenient and safe approach.

**Electronic supplementary material:**

The online version of this article (doi:10.1186/s12890-016-0318-x) contains supplementary material, which is available to authorized users.

## Background

Pleural effusions are a common clinical problem with more than 50 recognised causes [1]. In the UK, an estimated 50,000 diagnoses of MPE are made each year [2]. While fluid tumor markers may help in making a probable diagnosis of malignancy, they are not disease-specific [3], and cytological examination of pleural fluid for malignant cells establishes a positive diagnosis of malignancy in only 60% of carcinomatous effusions [4–6]. Immunostaining substantially improves the diagnostic yield [7] but this falls to 30% in effusions associated with malignant mesothelioma [8]. Thus, the role and value of fluid biomarkers and cytology are hotly debated [9].

The definitive diagnosis of pleural malignancy depends upon histological proof obtained via pleural biopsy. SPB, US-CNB and thoracoscopy are techniques commonly utilised for the acquisition of pleural tissue [10–15]. Thoracoscopy has a superior diagnostic yield for pleural effusions [16, 17] but it is relatively complicated to perform, especially in frail patients. With the lower diagnostic yields, SPB and US-guided CNB are now being neglected. However, given the ease of use of these procedure and their lesser costs, SPB or US-guided CNB may be considered the initial diagnostic step in undiagnosed pleural effusions. Currently, the most efficient and cost-effective approach for a definitive diagnosis remains difficult to establish and is controversial among chest physicians [18].

To our knowledge, no prospective studies have been undertaken to assess the utility of a combination of US-guided CNB and SPB performed sequentially in the same setting and by the same operator. Consequently, in this prospective study, we evaluated the value of a combination of US-guided CNB and SPB for diagnosis of MPEs.

## Methods

### Study design and setting

We conducted a prospective, non-randomised study at a dedicated respiratory centre (State Key Laboratory of Respiratory Disease and China Clinical Research Centre of Respiratory Disease, Guangzhou Institute of Respiratory Disease, Guangzhou).

### Patients

A total of 172 consecutive patients with pleural effusions who were treated at our institution between January 2013 and December 2014 were included in the study. The inclusion criteria for enrolment of patients were: (1) undiagnosed and untreated pleural effusion; (2) unilateral transudate as suggested by clinical images but unresolved upon treatment of the cause; and (3) age greater than 18 years. Exclusion criteria included: (1) bilateral pleural effusions; (2) minimal or small effusions; (3) insufficient bleeding diathesis for pleural aspiration and biopsy; and (4) an inability of the patient to provide written informed consent.

### Transthoracic ultrasound

All patients underwent initial conventional US scans (Esaote Mylab 90, Italy) without previous removal of pleural fluid. US was performed using splenic echotexture as an in vivo reference. The patients were in a sitting, prone, supine or lateral decubitus position when US was performed. They were divided into two groups: those exhibiting a maximum thickening of more than 3 mm, and those exhibiting a maximum thickening of less than 3 mm. The presence of effusion was confirmed by standard means, and the amount of effusion was documented as either minimal, small, moderate, or large [19]. All zones were screened, and the information obtained via US was used to select the entry site, route, sampling site, direction of biopsy and the biopsy depth. The lower thoracic parietal pleura close to the diaphragm was selected for biopsy unless other regions of the parietal pleura were thicker than the lower thoracic parietal pleura.

### Diagnostic thoracentesis and US-guided cutting-needle biopsy (CNB)

**Fig. 1:**
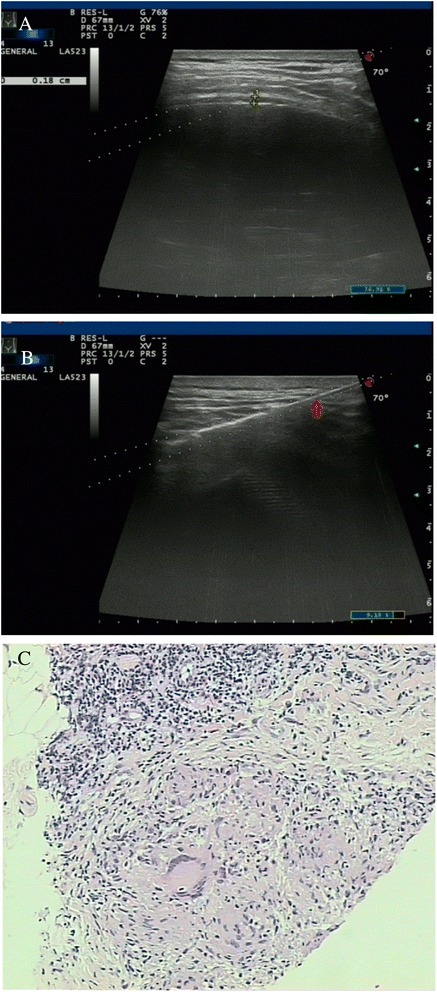
Images of a 42-year-old man with a history of shortness of breath for 1 month. **a** A conventional US scan showed an effusion and thickening of the parietal pleura (0.18 cm). **b** Real-time US-guided cutting-needle biopsy (arrowhead) focused on the pleura and was introduced at an angle of 70°. **c** A biopsy sample obtained from the pleura showed a tuberculoid nodule and caseous necrosis (H&E staining; magnification, × 10)

**Fig. 2:**
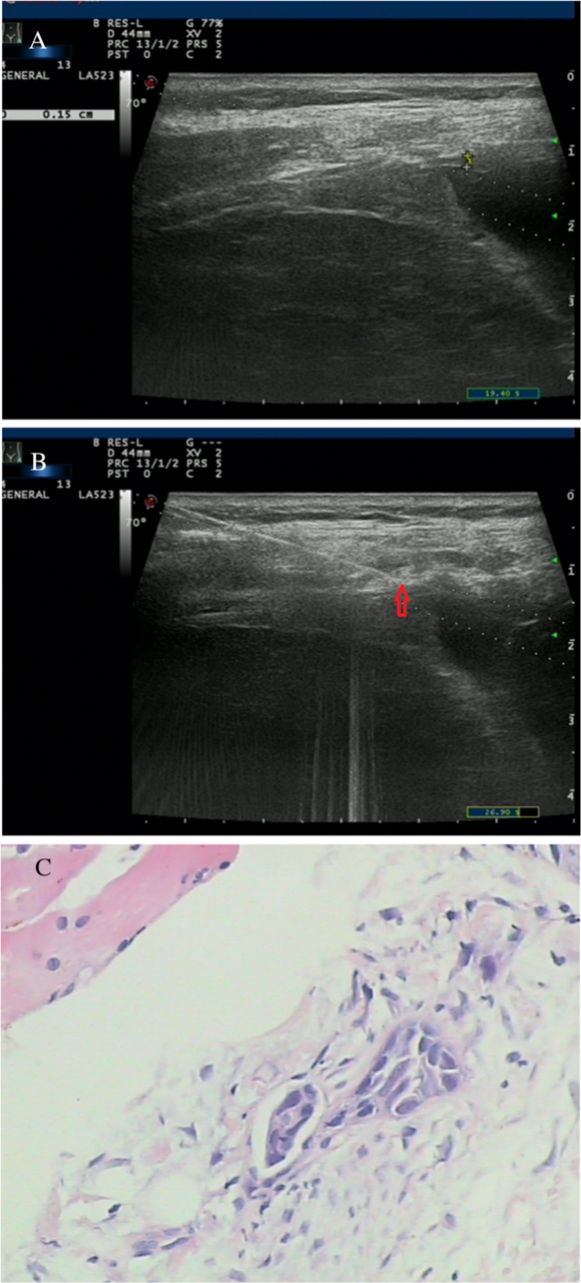
Images of a 54-year-old woman with a history of chest pain for 3 weeks. **a** A conventional US scan showed thickening of the lower thoracic parietal pleura close to the diaphragm (0.15 cm) with a low echo texture. **b** Real-time US-guided cutting-needle biopsy (arrowhead) focused on the pleura and was introduced at an angle of 70°. **c** Biopsy sample obtained from the pleura showed mesothelioma (H&E staining; magnification, × 100)

Prior to pleural biopsy, pleural effusions were collected from all subjects for biochemical and microbiological analyses, including pH, total protein, lactate dehydrogenases (LDH) and adenosine deaminase (ADA) levels. The biopsy procedures were performed under real-time visualisation using a 16-gauge spring-loaded automated cutting needle (MC1816, Bard Max-Core, Bard, Inc., USA) after thoracentesis. The cutting needle was inserted through the guiding channel and then introduced into the margin of the pleural area. At least four specimens were obtained from each patient, fixed in formaldehyde solution and transferred to the Pathology Department for histological examination and immunohistochemical analyses. One specimen was placed in a sterile tube and sent for mycobacterial culture. Figures 1 and 2 show the images obtained in two patients.

### Standard pleural biopsy (SPB)

Following US-guided CNB, SPB (Abrams’ biopsy) was performed at another site. For moderate effusions, the biopsies were obtained from the site exhibiting the maximum effusion as determined by US. In the case of large effusions, the puncture site was chosen to be as low as possible but not within 25 mm of the diaphragm. To acquire a sufficient number of specimens, we created one small incision (3–5 mm) on the skin; when necessary, the parietal pleura was pressed to establish complete contact with the biopsy needle. Collection of biopsy specimens with SPB was similar to that with US-guided CNB. In patients with large effusions, an indwelling pleural catheter was inserted after conducting all biopsy procedures to manage the subsequent steps for a definitive diagnosis. A routine follow-up chest x-ray was obtained within 24 h following the biopsy procedures to assess any possible complications.

All procedures were performed in a dedicated respiratory unit by an experienced physician (JW), and all US procedures were performed by the same experienced sonographer (XZ). The US patterns were evaluated by two observers (JW and XZ). Disputes regarding pleural areas were discussed until a consensus was reached. An experienced lung pathologist evaluated the biopsy specimens.

### Data analysis

A definitive diagnosis of pleural malignancy [true-positive (TP)] was made by histopathological analysis of the biopsy specimens, clinical follow-up and surgery, while a benign diagnosis [true-negative (TN)] was made if: (1) the benign histological diagnosis was based on a precise aetiology; (2) the pleural effusion subsequently disappeared; or (3) follow-up chest radiographs or computed tomography (CT) scans showed a small amount of pleural effusion that remained stable for at least 12 months. Patients with a benign histology were observed for 12 months to minimise the risk of potential false-negative (FN) results.

We combined the two biopsy methods for each patient and recognised a TP result if the two methods together or each of the methods individually showed a TP result. The patients’ clinical features, the characteristics of the parietal pleura, pathology reports on the biopsy specimens, the results of the cultured specimens, the definitive diagnoses, and clinical outcomes were all recorded.

### Statistical analysis

Data were reported as the number and percentage of qualitative variables. Enumerated data were presented as means ± standard deviation (SD). Categorical variables were analysed and statistical analysis was performed using SPSS® version 16.0 (IBM, Chicago, IL, USA). The primary endpoint was the sensitivity of each biopsy method (US-guided CNB or SPB) and the combination of the two methods for detection of pleural malignancy. Secondary endpoints were other elements of the decision matrix [(specificity, positive-predictive value (PPV), negative-predictive value (NPV) and diagnostic accuracy for pleural effusions)]. A χ^2^ test was used to compare the adequacy of biopsy specimens, diagnostic sensitivity, NPV and the diagnostic accuracy. Significance for all statistical analyses was set at *p* < 0.05.

## Results

### Characteristics of patients and transthoracic US

Of the 172 patients who were enrolled in this study, 20 had undergone a non-diagnostic pleural aspiration before visiting our institute, but none had previously undergone pleural biopsy procedures. Table 1 shows the demographic and pleural characteristics of the 172 patients; 80 exhibited moderate effusions while 92 had large effusions. Thoracic CT scanning or US were used to evaluate pleural thickening. Three patients had clear bulky nodules (between 18 and 25 mm thick on the CT scan), but 40 (23.3%) had no significant pleural thickening on the CT scan or US. Pleural thickness was less than 3 mm in112 patients and greater than 3 mm in 60 patients.

**Table 1:** Demographic and pleural characteristics of the patients

Parameter	Value
Number of patients	172
Sex (M/F)	108/64
Age, years (mean ± SD; range)	54.8 ± 5.8 (22–91)
Side of effusion (left/right)	96/76
Minimal effusions	0
Small effusions	0
Moderate effusions	80
Large effusions	92
Pleural thickness <3 mm	112
Pleural thickness ≥3 mm	60

Data are numbers of patients unless otherwise stated

### Definitive diagnosis of the pleural effusions

The definitive diagnosis in 90 of the 172 enrolled patients (52.3%) was pleural malignancy, while 82 (47.7%) had non-malignant disease as confirmed by the clinical follow-up (Table 2, Additional file 1: Excel). Two patients had identifiable micro-organisms in subsequent analyses of their biopsy specimens. In six cases, the material obtained with both biopsy techniques was inadequate; four of these patients suffered from a disease of indeterminate origin (as evidenced by more than 12 months of clinical follow-up), and the other two patients were diagnosed with lymphomas via thoracoscopy. Combined SPB and US-guided CNB revealed FN results in 10 cases. The final diagnoses for these patients were: mesothelioma in four (as revealed by thoracoscopy); adenocarcinoma in four [whose final diagnosis was determined by transbronchial lung biopsy (TBLB)]; and adenocarcinoma in two (who were also diagnosed with pleural tuberculosis which was progressive during follow-up when the final diagnosis was eventually made).

**Table 2:** Final diagnoses of the causes of pleural effusions in 172 patients

Malignant neoplasms	No.	Non-malignant disease	No.
Adenocarcinoma	42	Inflammatory pleuritis	16
Squamous cell carcinoma	12	Pleuritis fibrosis and plaques	6
Mesothelioma	10	Pleural tuberculosis	44
Lymphoma	4	Fungal infection	4
Pleural metastasis of breast cancer	4	Chronic empyema	6
Undifferentiated cell carcinoma	2	Indeterminate origin disease	4
Small lung cancer	16	Chronic heart failure	2

### Definitive diagnosis analyses

Adequate pleural biopsy specimens for histological analysis were obtained in 156 patients (90.7%) with US-guided CNB and 160 (93.0%) with SPB. The difference between the two techniques was not statistically significant (*p* = 0.577). When US-guided CNB was combined with SPB, adequate specimens were obtained in 166 patients (96.5%) using one or both techniques, but the number of specimens obtained was not significantly different from those obtained using US-guided CNB or SPB alone (*p* = 0.119 or 0.304).

The sensitivities of US-guided CNB, SPB and a combination of the two techniques for diagnosis of pleural malignancy were 51.2, 63.4 and 88.6%, respectively (Table 3). The combination of the two techniques significantly improved the sensitivity compared with each individual technique alone (*p* < 0.05), but there was no significant difference in sensitivity between US-guided CNB and SPB (*p* = 0.147). Significant differences in the NPV and diagnostic accuracy were also observed between the combination and the individual techniques alone (*p* < 0.05).

**Table 3:** Comparison of diagnostic accuracy between US-guided biopsy and standard biopsy

	CNB (*n* = 156)	SPB (*n* = 160)	CNB + SPB (*n* = 166)	Statistical significance
FN	40	30	10	NA
TN	74	78	78	NA
TP	42	52	78	NA
FP	0	0	0	NA
Sensitivity	51.2%	63.4%	88.6%	*p* = 0.147, 0.000, 0.000*
Specificity	100%	100%	100%	NA
PPV	100%	100%	100%	NA
NPV	64.9%	72.2%	88.6%	*p* = 0.394, 0.000, 0.009*
Diagnostic accuracy	74.4%	81.3%	93.9%	*p* = 0.341, 0.001, 0.017*

*CNB* cutting-needle biopsy, *FN* false-negative, *FP* false-positive, *NA* not applicable, *NPV* negative-predictive value, *PPV* positive-predictive value, *SPB* standard pleural biopsy, *TN* true-negative, *TP* true-positive, *US* ultrasound

*p-values for CNB vs SPB; CNB + SPB vs CNB; and CNB + SPB vs SPB

We also evaluated whether pleural thickness affected the diagnostic accuracy of the two biopsy methods. In patients with pleural thickening ≥3 mm, the diagnostic accuracy with US-guided CNB and SPB were 84.2 and 82.5%, respectively, and the difference between the two techniques was not statistically significant (*p* > 0.05). However, in the group with pleural thickening <3 mm, diagnostic accuracy was significantly greater with SPB than with US-guided CNB (*p* < 0.05).

With US-guided CNB, the diagnostic accuracy was significantly greater in patients with pleural thickening ≥3 mm in comparison with those with pleural thickening <3 mm (*p* < 0.05), but with SPB, there was no statistically significant difference the two pleural thickening groups (*p* > 0.05). The findings of this analysis are shown in Table 4.

**Table 4:** Diagnostic accuracy of the 2 biopsy techniques according to the degree of pleural thickening in US scans

Pleural thickening	CNB (*n* = 156)		SPB (*n* = 160)		*p*-Value
	No.	Accuracy (%)	No.	Accuracy (%)	
≥3 mm	57	49 (84.2)	57	47 (82.5)	0.607 (χ^2^ = 0.264)
<3 mm	99	67 (67.6)	103	83 (80.6)	0.036 (χ^2^ = 4.398)
*p*-Value		0.012 (χ^2^ = 6.345)		0.771 (χ^2^ = 0.085)	

*CNB* cutting-needle biopsy, *SPB* standard pleural biopsy

### Complications

The two biopsy procedures were generally well tolerated, and neither procedure was abandoned because of complications. With US-guided CNB, 8 of the 172 patients suffered from significant pain during the procedure and four of these patients required parenteral analgesics. Two moderate haemoptyses and two chest wall haematomas were observed in four patients following US-guided CNB, but none required further intervention. Following SPB, four patients experienced syncope, but none required any specific medical intervention and all recovered fully within 1 min. In addition, four patients suffered from a slight pneumothorax following SPB; these cases were suspected on the basis of US post-biopsy results and were confirmed by chest x-ray, which stopped spontaneously without treatment.

One patient who was diagnosed with a mesothelioma showed an implantation metastasis in the CNB incision after 3 months of follow-up.

## Discussion

The definitive diagnosis of pleural diseases, particularly malignancy, depends upon histological analysis of tissue obtained via pleural biopsy. Adequate pleural tissues, which are crucial for a definitive diagnosis, can be obtained by SPB, thoracoscopy or CNB under the guidance of CT or US. Thoracoscopy allows direct visualisation of the pleura and biopsy from abnormal sites [20]. In a study of patients with pleural tuberculosis, Koegelenberg et al [21]. found that US-assisted Abrams’ needle biopsy specimens were more likely to contain pleural tissue than specimens obtained using US-assisted Tru-Cut biopsies (91.0 vs 78.7%; *p* = 0.015). In 2014, Hallifax et al [22]. reported that US-guided CNB successfully obtained pleural tissue in a high proportion of patients (94.0%) with pleural disease, including cases where thoracoscopy had failed. The present study is the first prospective investigation of a combination of US-guided CNB and SPB for the diagnosis of MPE. Our results showed that US-guided CNB and SPB provided adequate specimens for histological analysis in 90.7 and 93.0% of cases, respectively (*p* = 0.577), while the combination of both techniques provided adequate specimens in 96.5% of cases; however, the latter result was not significantly superior to the two techniques alone (*p* = 0.119 or 0.304).

Current guidelines on the investigation of pleural effusions emphasise the use of a diagnostic algorithm or recommend the use of a stepwise approach [6, 23–25]. However, pleural effusion analyses and biomarkers are not disease-specific [3, 26–28]. Previous biopsy investigations have mostly focused on the advantages and limitations of each individual technique, and to our knowledge, no studies have investigated a combination of CNB and SPB at the same time. Our results indicate that a combination of the two techniques is more effective than either technique alone for the diagnosis of malignant pleural disease. The size of this advantage is considerable. The combination of CNB and SPB led to a correct diagnosis of MPE in 88.6% of patients, and the sensitivity (88.6%) was only slightly lower than published sensitivities from large thoracoscopy series [29, 30]. Thoracoscopy has the advantage of undertaking some therapeutic options, such as talc poudrage, at the same time, but has the disadvantages of being more costly, more invasive, and hazardous in very frail patients. The combination of CNB and SPB performed sequentially by same operator in the same setting can avoid the need for repeated procedures (since the sensitivities of the combination of US-guided CNB and SPB and the two techniques alone for diagnosis of pleural malignancy were 88.6, 51.2 and 63.4%, respectively, 37 or 25% of patients compared with CNB or SPB alone could avoid the need for repeated procedures), and it would decrease both medical costs and the time required for evaluation of pleural malignancy.

SPB was described more than 50 years ago and became the most widely utilised method for blind biopsy [31]. This procedure has some advantages, including a relatively low cost and ease of usage, but it generally demonstrates a modest diagnostic accuracy of less than 60% for MPE [5, 6],although a higher diagnostic accuracy for pleural tuberculosis (80–87%) [17, 32]. In recent years, US-guided CNB has been increasingly used for pleural biopsy. The most obvious advantage of this procedure is its ability to ensure that biopsy samples are obtained from areas characterised by abnormal pleural tissue. While US-guided CNB increases the diagnostic accuracy and minimises the risk compared with SPB [33, 34], its diagnostic accuracy is lower than that of thoracoscopy [6, 35]. However, the use of thoracoscopy is not always possible in frail patients or when pleural fluid is heavily loculated or the lung is adherent to the chest wall.

To overcome these limitations, a combination of CNB and SPB was used in this study. All procedures were performed sequentially by an experienced operator (JW) according to standardised guidelines. Several possible factors could be responsible for the diagnostic advantage of the combination in comparison with the individual techniques. During SPB, an incision in the skin in the direction of the chosen intercostal space above the lower rib was made, especially in overweight/obese patients. During the biopsy procedure, the assistant pressed the skin between the ribs, which allowed the distal tip of the needle to have sufficient contact with the pleura. A limitation of SPB is the blindness of the procedure, although an experienced operator can obtain adequate tissues for a histological diagnosis. We found that the number of adequate specimens was higher with SPB than with CNB (93.0 vs 90.70%, respectively), and, as previously reported [17, 32–36], the diagnostic accuracy was also higher (81.3 vs 74.4%, respectively). In addition, for patients with a pleural thickness <3 mm, the diagnostic accuracy of SPB was also significantly higher than with CNB (*p* < 0.05).

For CNB, we performed the procedures using relatively supradiaphragmatic biopsy sites or the most thickened pleural sites. Pleural malignancy is characteristically patchy and preferentially basal, or is found on the diaphragm and results in focal involvement [36]. In addition, a large angle may be essential to obtain adequate samples, especially from thin pleura. The cutting needle was cautiously introduced at an angle of more than 55° through an incision in the skin made toward the direction of the chosen intercostal space under the guidance of a high-frequency probe. However, as has previously been reported [17, 32–36], our results showed that the diagnostic accuracy of CNB was lower than that of SPB (74.4 vs 81.3%, respectively). When our results were analysed according to the degree of pleural thickening, the diagnostic accuracy of CNB in patients with pleural thickening <3 mm was significantly lower than in patients with pleural thickening ≥3 mm (*p* < 0.05), and significantly lower than with SPB (*p* < 0.05). A possible reason for this finding may be that the diagnostic accuracy is affected by pleural thickening. When a US-guided pleural biopsy is performed in patients with minor pleural thickening, there may be a lower probability of obtaining adequate specimens. However, SPB may be capable of acquiring a larger number of adequate samples.

Both biopsy procedures were well tolerated in the patients we studied, and no serious complications were observed. However, we concerned with that although four significant pain and two moderate haemoptysis required no intervention, there were about 3.5% complications (four significant pain and two chest wall haematoma) required further intervention following CNB. Following SPB, four patients (2.3%) suffered from a slight pneumothorax, but which recovered spontaneously. Though neither procedure was abandoned for the complication, management must be to improve to avoid it, such as, better preparation for reducing syncope or significant pain, skilled procedures for avoiding haemoptysis, chest wall haematoma or pneumothorax. Reported complication rates of SPB or image-guide CNB vary widely [16, 37, 38]. There was 11% had a new pneumothoraces visible on CT following CT-guided CNB [38], but major complication was rare. Thoracoscopy has demonstrated a low rate of complications, but mortality rates resulting from major complications (including air leak and pneumonia) have been reported to be 0.34–1.8% [2, 39]. So, the combined approach was safe. In addition, compared with the suggested stepwise approach and thoracoscopy [6, 16], the combination was finished in the same operation sequentially, it shortened the days of hospitalization, decreased the cost and it was convenience. In our study, one patient was found to have an implantation metastasis at the biopsy site after 3 months of follow-up. Whether prophylactic radiotherapy of the site can reduce the likelihood of this complication is controversial [4, 40].

## Conclusions

Use of a combination of US-guided CNB and SPB afforded a high sensitivity to diagnose MPEs, it is a convenient and safe approach.

## Additional file

Additional file 1: Excel: Detailed results of CNB, SPB, CNB or SPB of the 17 2 UPE patients. (XLS 33 kb)

## Abbreviations

ADA: Adenosine deaminase
CNB: Cutting-needle biopsy
CT: Computed tomography
FN: False-negative
LDH: Lactate dehydrogenase
MPE: Malignant pleural effusion
NPV: Negative-predictive value
PPV: Positive-predictive value
SD: Standard deviation
SPB: Standard pleural biopsy
TBLB: Transbronchial lung biopsy
TN: True-negative
TP: True-positive
US: Ultrasound
